# Involvement of TRPA1 receptors in meningeal blood flow induced by formation of nitroxyl (NO-/HNO)

**DOI:** 10.1186/1129-2377-14-S1-P88

**Published:** 2013-02-21

**Authors:** M Dux, M Eberhardt, MR Filipovic, R DeCol, K Messlinger

**Affiliations:** 1 Department of Physiology, University of Szeged, Hungary; 2 Institute of Physiology & Pathophysiology, University of Erlangen-Nuernberg, Germany; 3 Department of Chemistry and Pharmacy, University of Erlangen-Nuernberg, UK

## Introduction

Activation of TRPA1 receptors has recently been shown to cause release of calcitonin gene-related peptide (CGRP) from trigeminal ganglion neurons and to increase meningeal blood flow in animals ( Kunkler et al. 2011 ), events regarded to be associated with headaches. Meningeal blood flow is also increased by donors of nitric oxide (NO), partly mediated by the release of CGRP from meningeal afferents ( Strecker et al. 2002 ).

## Objective

The release of CGRP provoked by NO donors may be indirectly induced by the formation of nitroxyl (NO-/HNO), a reduced congener of NO ( Switzer et al. 2009 ), which may activate TRPA1 receptors of meningeal afferents. Considering this possibility we examined the role of NO-/HNO in a rat model of meningeal blood flow.

## Methods

In isofluorane anaesthetised rats, meningeal blood flow was recorded by laser Doppler flowmetry. Sodium-alpha-oxyhyponitrite (Angeli’s salt, AS, 300 µM), which mainly produces NO-/HNO, was topically applied to the cranial dura mater. The distribution of TRPA1 immunoreactive neurons in the trigeminal ganglion was determined by indirect immunohistochemistry.

## Results

Application of AS caused increases in meningeal blood flow lasting several minutes. Topical pre-administration of 50 µM HC-030031, a specific inhibitor of TRPA1 receptors, reduced the blood flow increases to the half. Topical applications did not change arterial blood pressure and heart rate. TRPA1 receptor immunoreactivity was found in a proportion of small trigeminal ganglion neurons.

## Conclusions

We conclude that NO-/HNO can increase meningeal blood flow by activating TRPA1 receptors, most likely through stimulation of CGRP release from meningeal afferents. Similar mechanisms may be involved in the pathophysiology of headaches associated with the endogenous NO metabolism.
